# Acute Toxicity and the Effect of Andrographolide on *Porphyromonas gingivalis*-Induced Hyperlipidemia in Rats

**DOI:** 10.1155/2013/594012

**Published:** 2013-06-13

**Authors:** Rami Al Batran, Fouad Al-Bayaty, Mazen M. Jamil Al-Obaidi, Mahmood A. Abdulla

**Affiliations:** ^1^Center of Studies for Periodontology, Faculty of Dentistry, University Technology MARA (UiTM), 40450 Shah Alam, Selangor, Malaysia; ^2^Department of Biomedical Science, Faculty of Medicine, University of Malaya, 50603 Kuala Lumpur, Malaysia

## Abstract

The aim of the current study is to evaluate the effect of andrographolide on hyperlipidemia induced by *Porphyromonas gingivalis* in rats. Thirty male *Sprague Dawley* (SD) rats were divided into five groups as follows: group 1 (vehicle) and four experimental groups (groups 2, 3, 4, and 5) were challenged orally with *P. gingivalis* ATCC 33277 (0.2 mL of 1.5 ×10^12^ bacterial cells/mL in 2% carboxymethylcellulose (CMC) with phosphate-buffered saline (PBS)) five times a week for one month to induce hyperlipidemia. Then, group 3 received a standard oral treatment with simvastatin 100 mg/kg, and groups 4 and 5 received oral treatment with andrographolide 20 mg/kg and 10 mg/kg, respectively, for another month. The results showed that total cholesterol (TC), low-density lipoprotein (LDL-C), and triglycerides (TG) were reduced significantly in groups treated with andrographolide. The malondialdehyde (MDA) level was low in treated groups, while antioxidant enzymes, superoxide dismutase (SOD), and glutathione peroxidase (GPx) were significantly increased in these groups (P < 0.05). Liver tissues of the groups treated with andrographolide reduce the accumulation of lipid droplets in hepatic tissue cells. An acute toxicity test did not show any toxicological symptoms in rats.

## 1. Introduction

The degradation of periodontal tissues in periodontal disease results from inflammatory reactions primarily derived from interactions between the host's immune system and subgingival bacterial colonies [1]. Degradation is thought to be caused by the buildup of microorganisms on the tooth surface in periodontal pockets. In spite of the more than 500 diverse bacterial species present in the oral cavity, only a relatively small number of organisms are associated with periodontal ailments. On the other hand, *Porphyromonas gingivalis* is the most widespread organism connected to mature types of periodontal infection. Over the past ten years, growing proof has supported the role of periodontal illness and infection with *P. gingivalis *as a possible contributor to a number of systemic sicknesses, such as diabetes, preterm birth, heart disease, and atherosclerosis [2]. *P. gingivalis*, a black-pigmented gram-negative anaerobic bacterium, is the most significant organism contributing to mature periodontitis [3]. *P. gingivalis *has a number of bioactive molecules on its cell surface that contribute to its pathogenesis, such as cytoplasmic coverings, peptidoglycans, outer casing proteins, lipopolysaccharides (LPS), pills, caspases and fimbriae, which can induce excessive cytokine production.* P. gingivalis *LPS is a vital pathogenic part of the commencement and growth of periodontal infections [4].

*Andrographis paniculata *(Burm.f.) Nees (Acanthaceae) is a long-established therapeutic plant in Southeast Asia that originated in India and Indo-China. The plant is generally recognized as the “king of bitter.” *A. paniculata *contains andrographolide as the main dynamic code as well as other codes such as 14-deoxy-11, 12-didehydroandrographolide, and 14-deoxyandrographolide. The plant is also known for its hypotensive [5], anti-hyperglycemic [6], phagocytic, and antibacterial activities [7], wound healing [8], gastroprotective [9], and antithrombotic and anticancer properties [10]. The present study intended to assess hypolipidemic and antioxidant properties of andrographolide in rats.

## 2. Materials and Methods

### 2.1. Chemicals

Andrographolide was purchased from Sigma-Aldrich (USA) and* Porphyromonas gingivalis *from ATCC (USA).

### 2.2. Acute Toxicity Study

An acute toxicity study was used to determine a safe dose of andrographolide. Healthy male and female *Sprague Dawley* rats (6–8 weeks old) were acquired from the Animal House (University Technology Mara, Ethic number 28/05/2012. 600-FF (PT. 5/2)). The rats weighed between 180 and 200 g. The rodents were fed with normal rat pellets and tap water *ad libitum *and were individually placed in separate cages with wide-mesh wire bottoms to avert coprophagia for the duration of the test. Thirty-six rats (18 males and 18 females) were assigned uniformly to three groups tagged as vehicle (0.5% CMC, 5 mL/kg), 100 mg/kg and 500 mg/kg of andrographolide (5 mL/kg). The rats were subjected to fasting throughout the night (food but not water was absent) before dosing. Food was withheld for an additional 3 to 4 hours following dosing. The rodents were monitored for 48 hours following treatment for the observation of medical or toxicological symptoms. Death was monitored for a period of more than two weeks. The animals were sacrificed using an overdose of xylazine and ketamine anesthesia on the 15th day. Histological, hematological, and serum biochemical profiles were established using normal methods [11]. Throughout the tests, all rodents were given gentle care in line with the criteria stipulated in the “Guide for the Care and Use of Laboratory Animals” [12].

### 2.3. Bacterial Culture

Under anaerobic conditions, *P. gingivalis* ATCC strain 33277 was cultured on anaerobic blood agar plates (Becton Dickinson Co.) in an aerobic chamber (Coy Laboratory Products, Inc.) with 85% N_2_, 5% H_2_, and 10% CO_2_ for 3 to 5 days and then inoculated into Schaedler Broth (Difco Laboratories) containing hemin and menadione for 24 hours, according to the previous protocol with some modifications [13].

### 2.4. Animals and Experimental Design

Healthy male *Sprague Dawley* rats (6–8 weeks old) were acquired from the Animal House, (University Technology Mara, Ethic number 28/05/2012. 600-FF (PT. 5/2)). In this study, thirty rats were maintained on a twelve-hour day/night cycle at an ambient temperature with free access to a normal rat diet along with tap water *ad libitum, *and each group was placed in a separate cage. Rats were divided into 5 groups of 6 rats per cage as follows: normal (1) and experimental groups (2, 3, 4, and 5). Experimental groups were given antibiotics in daily dose of ampicillin/kanamycin (2 mg per drug per day) administered orally for ten days and were then challenged orally with *P. gingivalis *ATCC 33277 (0.2 mL of 1.5 × 10^12^ bacterial cells/mL in 2% CMC with PBS) five times a week for one month to induce hyperlipidemia. Then, groups 3, 4, and 5 were given simvastatin (100 mg/kg), andrographolide(20 mg/kg, 10 mg/kg), respectively, daily for one month [14, 15].

### 2.5. Biochemical Analysis

Blood samples were collected and centrifuged at 3500 g for 15 min to obtain serum. The levels of serum total cholesterol (TC), triglycerides (TG), low-density lipoprotein-cholesterol (LDL-C), high-density lipoprotein-cholesterol (HDL-C), and liver and renal functions in the serum were analyzed at the University of Malaya Medical Centre to evaluate the changes in biomarkers using a Hitachi Autoanalyzer, Japan.

### 2.6. Assay of Lipid Peroxidation

To evaluate the level of lipid peroxidation in serum, the level of malondialdehyde was estimated using thiobarbituric acid-reactive substances as indicators of lipid peroxidation, according to the procedure reported by Draper and Hadley with some modifications [16]. The serum was mixed with a solution (0.125 mL) containing 26 mM thiobarbituric acid, 0.26 M HCl, 15% trichloric acid, and 0.02% butylated hydroxytoluene. The mixtures were heated at 96°C for 15 minutes and centrifuged at 4,000 rpm for 10 minutes. The supernatant was transferred to 96-well plates, and the absorption was measured at 532 nm using tetramethoxypropane as the standard.

### 2.7. Antioxidant Enzymes

Superoxide dismutase (SOD) and glutathione peroxidase (GPx) were determined using commercial kits (Cayman-Michigan, USA), according to the manufacturer's instructions.

### 2.8. Hepatic Morphology

Livers were removed from the rats and put in a buffer solution of 10% formalin. Fixed tissues were processed routinely for paraffin embedding, and 4 *μ*m sections were prepared and dyed with hematoxylin and eosin stain.

### 2.9. Statistical Analysis

All values were reported as the mean ± SEM. Data were analyzed by one-way ANOVA and Dunnett's post hoc test for multiple comparisons using SPSS 18 software. Significance was defined as *P* < 0.05 compared to G2.

## 3. Results

### 3.1. Acute Toxicity

**Figure 1:**
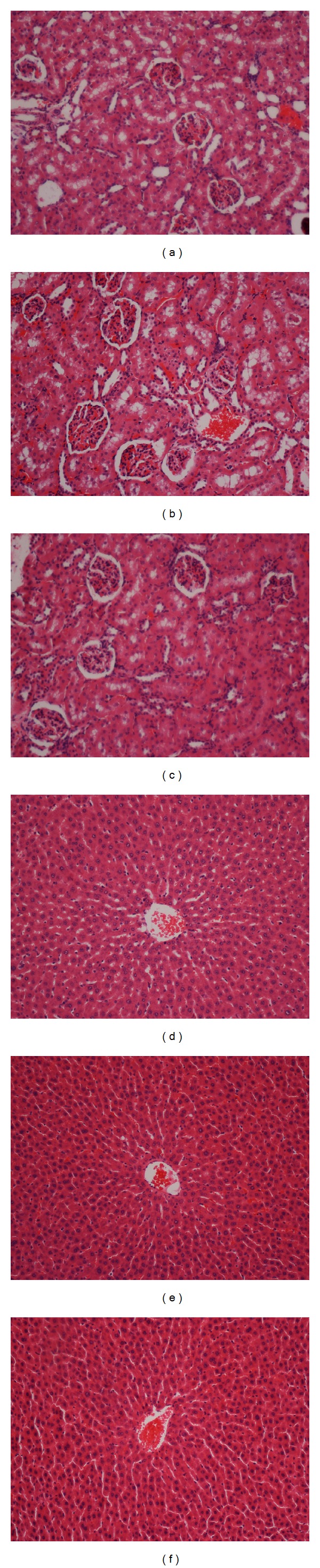
Effect of andrographolide on histological sections of the liver and kidney. Histological sections of the kidney (first row) and liver (second row) in an acute toxicity test. ((a) and (d)) Section from rats treated with CMC. ((b) and (e)) Sections from rats treated with 100 mg/kg andrographolide. ((c) and (f)) Sections from rats treated with 500 mg/kg andrographolide. There are no differences in the structures of the livers and kidneys between the treated and control groups (H&E staining, 20x).

All treated animals were closely observed for any behavioral changes, abnormal, or toxic manifestations and for mortality up to 24 h. There was no mortality that occurred in the treatment group (andrographolide with dose levels of 100 and 500 mg/kg body weight) during the study period. Renal (sodium, potassium, chloride, CO_2_, anion gap, urea and creatinine) and liver (total protein, albumin, globulin, total bilirubin, conjugated bilirubin, Alkaline phosphatase, alanine aminotransferase, aspartate aminotransferase, and gamma-glutamyltransferase) biochemical parameters did not show any significant differences of andrographolide-treated groups compared to control group in male and female rats (see the supplementary data in Supplementary Material available online at http://dx.doi.org/10.1155/2013/594012). On the other hand, histological evaluation of kidney and liver did not show any damage in the tissue (Figure 1). Acute toxicity studies showed that the compound was found to be safe up to a maximum dose of 500 mg/kg body weight of the animal.

### 3.2. Effect of Andrographolide on Blood Lipids

Variations in the overall serum lipid parameters were monitored in the control and treated groups.  Table 1 shows that there were significant increases in total serum cholesterol (TC), low-density lipoprotein (LDL-C), high-density lipoprotein (HDL-C), and triglycerides (TG) in the rats challenged only with *P. gingivalis *(group 2). Those biomarkers were significantly decreased in groups treated with andrographolide 10 mg/kg and 20 mg/kg (groups 4 and 5).

### 3.3. Effects of Andrographolide on Biochemical Analyses (Renal and Liver Biomarkers)

There were no significant differences in the levels of potassium (K), urea, and creatinine of the treated groups compared to control group, while sodium (Na) was significantly increased in treated groups compared to group 2 (Table 2).

The liver enzyme activities shown in Table 3 indicate that there was no considerable disparity in the total bilirubin (TB) level. Furthermore, alkaline phosphatase (AP) and alanine aminotransaminase (ALT) were elevated in the serum of group 2 (challenged only with *P. gingivalis*) and lower in the treated groups (groups 4 and 5). On the other hand, albumin and aspartate aminotransferase (AST) were elevated in the treated groups and lower in group 2.

### 3.4. Effects of Andrographolide on Lipid Peroxidation Level (MDA) and Antioxidant Enzymes (SOD and GPx)

**Figure 2:**
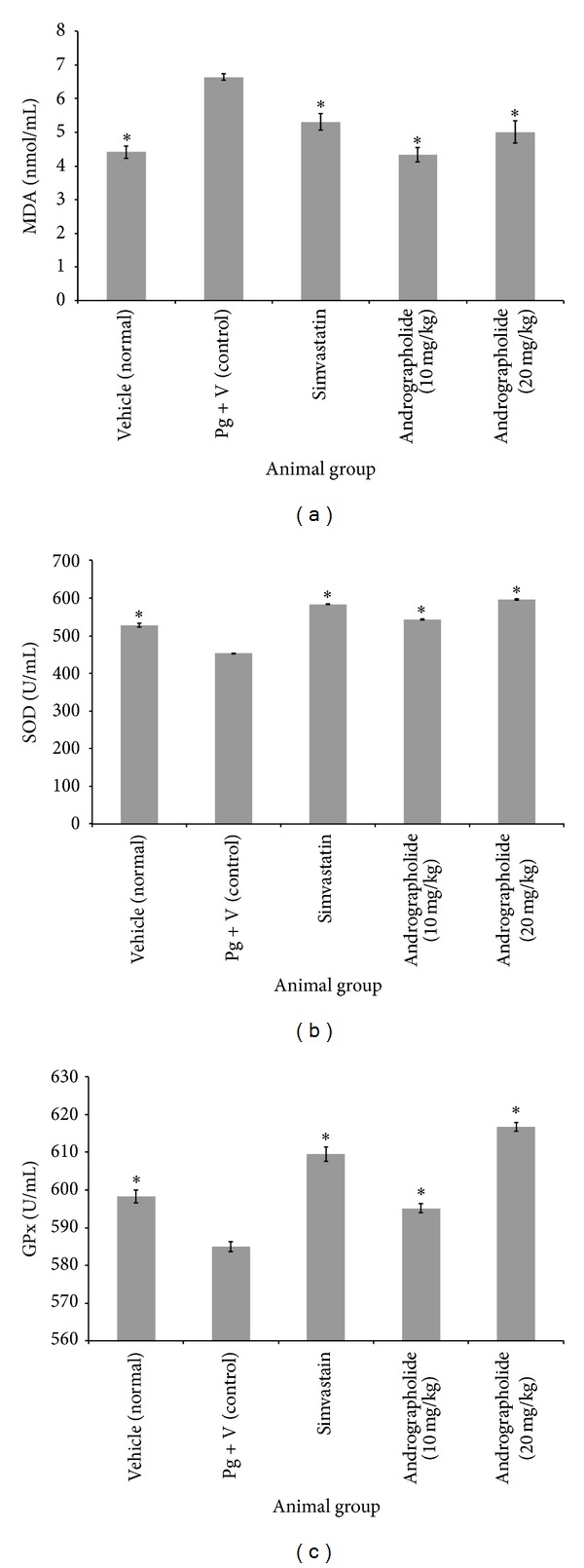
Effect of andrographolide on (MDA, SOD, and GPx) activity in serum in hyperlipidemic rats. (a) Effect of andrographolide on (MDA) malondialdehyde (*F*  (4,25) = 16.8, *P* < 0.001), (b) (SOD) superoxide dismutase (*F*  (4,25) = 485.5, *P* < 0.001), and (c) (GPx) glutathione peroxidase (*F*  (4,25) = 69.9, *P* < 0.001). Statistical analysis of the data was carried out using a one-way analysis of variance (ANOVA) and Dunnett's post hoc test for average comparison on SPSS 18.0. Mean values ± SEM (*n* = 6) were used. Significance was defined as *P < 0.05 compared to control group.

In the present study, malondialdehyde (a byproduct of lipid peroxidation) was analyzed in the serum of hyperlipidemic rats. Plasma lipid peroxidation concentrations were increased in group 2, while the treated groups showed a significant decrease in plasma lipid peroxidation levels (Figure 2(a)). The effect of andrographolide in hyperlipidemic serum on SOD and GPx levels is shown in Figures 2(b) and 2(c), respectively. SOD and GPx levels were elevated in the treated groups with andrographolide and lower in group 2.

### 3.5. Histological Evaluation of Liver Tissues

**Figure 3:**
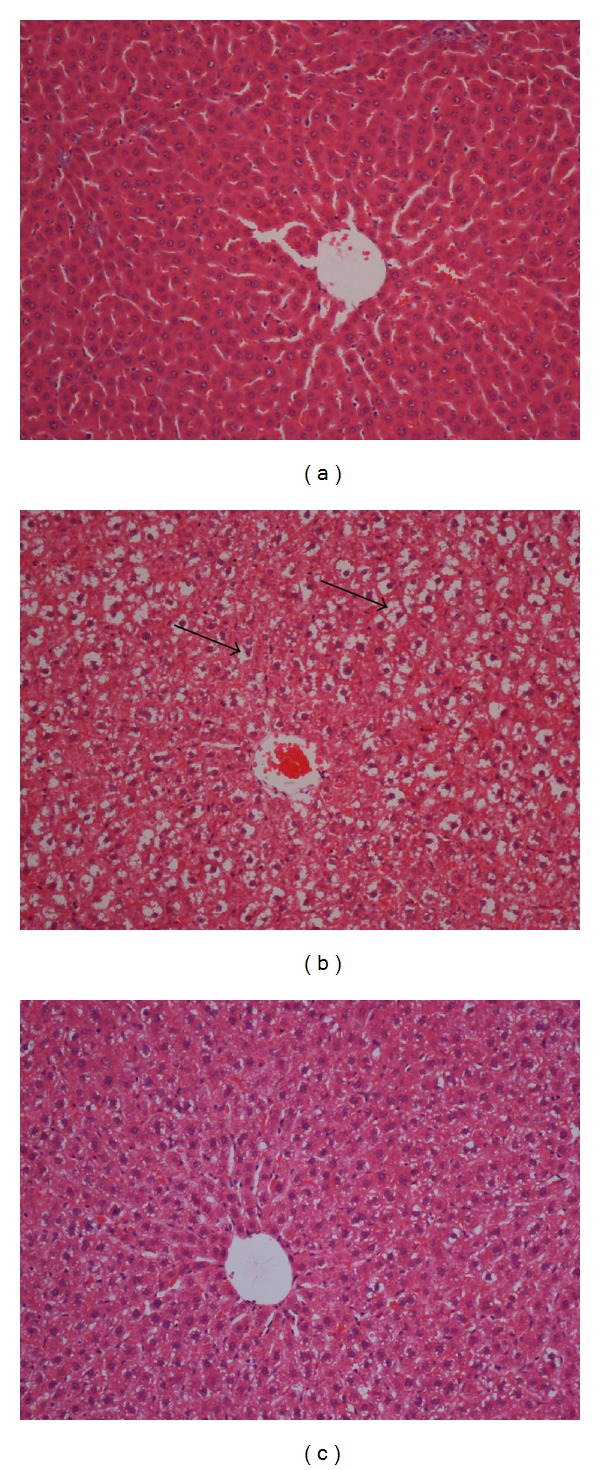
Effect of andrographolide on liver tissue morphology in hyperlipidemia rats. (a) No fat droplets were found in the liver of normal control group; (b) many large fat droplets were indicated by the arrowheads in the liver of rats challenged orally with *P. gingivalis;* (c) lower accumulation of lipid droplets shown in hepatic tissue of treatment group with 20 mg/kg andrographolide (H&E staining, 20x).

Effects of andrographolide on liver tissues are shown in Figure 3. The liver histological examination of the normal group showed normal cell architecture, while the liver histological examination of the negative control group (challenged only with *P. gingivalis*) showed remarkable morphological changes (less cells and lipid vacuolization). Liver tissues of the treatment group with 100 and 200 mg/kg andrographolide reduced the accumulation of lipid droplets in hepatic tissue cells.

## 4. Discussion

To determine the safety of drugs and plant products for human use, toxicological evaluation is carried out in various experimental animals to predict toxicity and to provide guidelines for selecting a “safe” dose in humans. Liver and kidney of the tested rats showed no significant change as compared to the control group. Clinical biochemistry values were within the range of the control animals tested. The highest dose of andrographolide, which did not cause any toxicity, was 500 mg/kg body weight, suggesting that this compound is relatively nontoxic since in acute toxicity studies, no deaths or any clinical signs of toxicity are observed.

There is strong evidence that *P. gingivalis* may play a significant role in the development of atherosclerotic lesions. In this study, the hypolipidemic and antioxidant effect of andrographolide in hyperlipidemic rats was investigated. To induce hyperlipidemia in rats, *P. gingivalis* feed was associated with PBS and CMC for one month. 

Reduction in the lipid profile of groups treated with andrographolide was in accordance with the results obtained by Choudhary et al. [17] who showed that ethanolic extract of *Iris germanica* significantly lowered the lipid components, especially cholesterol and triglycerides. An increase in LDL-C has been found to be one of the risk factors for the development of atherosclerosis and related cardiovascular diseases. Hence, the results obtained in this study indicated that hyperlipidemia indicators have been highly remarkable in the control group and decreased in treated groups; therefore, the results reflect the hypolipidemic effect of andrographolide in reducing the levels of the lipid profile, which could be due to the antioxidant properties of andrographolide. Furthermore, enhanced serum levels of AST and ALT would occur in the case of liver injury, which were specific toxicological indexes for liver function [18]. The serum ALT activities in the treated groups were significantly lower than in the control group (*P* < 0.05). In other words, andrographolide causes no toxicity to the liver function and minimizes the injury caused by *P. gingivalis*, so it could be concluded that andrographolide administration had a liver protective activity. 

Alanine aminotransaminase (ALT) was elevated in the control group and lowered in the groups treated with andrographolide. These results were similar to Bolkent et al. [19] who reported an increase in the levels of serum cholesterol (ALT). Therefore, these findings could justify the role of andrographolide in the treatment of hyperlipidemia in SD rats. The elevated serum activity of these enzymes has been reported as an indicator of cardiovascular disease. On the other hand, elevated serum ALT levels in the absence of viral hepatitis and alcoholism have been reported to lead to a higher risk of cardiovascular disease.

In the present study, plasma lipid peroxidation concentrations were lowered in the groups treated with andrographolide that were similar to the findings of Hou et al. [20]. The increase of MDA in the control group confirmed the increase of serum cholesterol and LDL levels. The increase of serum MDA causes a reduction in the antioxidant levels. The absence of the antioxidant property of andrographolide in the control group can explain the reverse relationship between antioxidant and serum MDA levels, which may be due to increased oxidative stress from various sources or a decrease in the antioxidant mechanism. It is possible that an antioxidant decrease was responsible for the diminished antioxidant activity.

Superoxide and hydroxyl radicals are produced in living systems and elaborate systems of defense and repair, which minimize the destruction of these reactive species. Therefore, it is possible that the increased serum SOD activity in treatment groups was sufficient to completely remove the formed superoxide anion radicals compared to the negative control group. An inversion phenomenon was observed by Mantha et al. [21] in hypercholesterolemic rabbits. In the present study, the activity of antioxidant enzyme GPx was increased in serum of treatments groups. Iliskovic and Singal [22] used adriamycin as a treatment agent, which decreased glutathione peroxidase activity and increased lipid peroxidation. However, because the decreased activities of SOD and GPx were associated with enhanced MDA levels in the negative control group, this suggests that stimulation of the enzymatic antioxidant defense system was not adequate to prevent lipid peroxidation. These results demonstrated the mechanism through which andrographolide prevents and reduces hyperlipidemia by enhancing enzymatic antioxidant secretion in the treatment groups. On the other hand, it could be due to the antioxidant properties of andrographolide ability to form antioxidants molecules, which can safely interact with free radicals and terminate the chain reaction before the vital molecules are damaged. Del Boccio et al. [23] reported that the activities of aortic SOD and GPx increased in cholesterol-fed rabbits compared to the control.

Liver histological examination of the normal group showed normal cell architecture, while significant morphological changes were observed in the group challenged with *P. gingivalis,* which showed fewer cells and lipid vacuolization. On the other hand, accumulation of hepatic lipid droplets appeared to be relatively lower in treatment groups. These results seemed to correspond to the serum lipid profiles and were consistent with previous studies [18]. Our study demonstrated that andrographolide could reduce the accumulation of lipid droplets in hepatic tissue cells of hyperlipidemia rats and prevent cardiovascular disease.

## 5. Conclusions

Andrographolide showed a hypolipidemic effect and significantly reduced the lipid profile levels and liver enzymes (AST and ALT). On the other hand, andrographolide improved enzymatic activity (SOD and GPx) and further decreased lipid peroxidation in the treated group. Andrographolide reduced the accumulation of hepatic lipid droplets in treatment groups, which reflect the potent antioxidant properties of andrographolide.

## Supplementary Material

No signs of any significant abnormality were observed in renal or liver observations at doses 100 mg/kg and 500 mg/kg of andrographolide for 15 days between the control and treated groups.

## Figures and Tables

**Table 1:** Effect of andrographolide on blood lipids in hyperlipidemic rats.

	TC (mmol/L)	LDL-C (mmol/L)	HDL-C (mmol/L)	TG (mmol/L)
Group 1	1.55 ± 0.02*	1.48 ± 0.04*	0.085 ± 0.002*	0.483 ± 0.04
Group 2	1.8 ± 0.00	1.63 ± 0.01	0.147 ± 0.004	0.55 ± 0.02
Group 3	1.35 ± 0.02*	1.33 ± 0.04*	0.088 ± 0.003*	0.28 ± 0.03*
Group 4	1.78 ± 0.01*	1.38 ± 0.01*	0.093 ± 0.033*	0.25 ± 0.02*
Group 5	1.33 ± 0.01*	1.24 ± 0.01*	0.085 ± 0.002*	0.35 ± 0.02*

(TC) Total cholesterol; *F* (4,25) = 137.7, *P* < 0.001, (LDL-C) low-density lipoprotein; *F* (4,25) = 33.49, *P* < 0.001, (HDL-C) High-density lipoprotein; *F* (4,25) = 72.6, *P* < 0.001, (TG) triglycerides; *F* (4,25) = 20.5, *P* < 0.001.

Group (1) vehicle; Group (2) vehicle and *P. gingivalis*; Group (3) simvastatin (100 mg/kg); Group (4) andrographolide 20 mg/kg; Group (5) andrographolide 10 mg/kg. Statistical analysis of the data was carried out using a one-way analysis of variance (ANOVA) and Dennett's post hoc test for average comparison on SPSS 18.0. Mean values ± SEM (*n* = 6) were used. Significance was defined as *P* < 0.05 compared to G2. Values without an asterisk do not have significant difference compared to G2.

**Table 2:** Effect of andrographolide on kidney biomarkers in hyperlipidemic rats.

	Na (mmol/L)	K (mmol/L)	Urea (mmol/L)	Creatinine (*µ*mol/L)
Group 1	143.17 ± 0.4*	4.47 ± 0.1	10.5 ± 0.24	38 ± 2.45
Group 2	138.67 ± 0.33	4.13 ± 0.07	11.97 ± 0.29	45.5 ± 0.22
Group 3	143.5 ± 0.34*	4.32 ± 0.05	12.62 ± 0.39	39.5 ± 2.69
Group 4	144.5 ± 0.34*	4.7 ± 0.18*	11.98 ± 0.46	43.17 ± 2.82
Group 5	146.17 ± 0.4*	4.47 ± 0.08	11.67 ± 0.55	41.17 ± 0.54

Sodium (Na); *F* (4,25) = 58.4, *P* < 0.001, Potassium (K); *F* (4,25) = 3.9, *P* < 0.05, Urea; *F* (4,25) = 3.7, *P* < 0.05, Creatinine; *F* (4,25) = 2.0, *P* = 0.119.

Group (1) vehicle; Group (2) vehicle and *P. gingivalis*; Group (3) simvastatin (100 mg/kg); Group (4) andrographolide 20 mg/kg; Group (5) andrographolide 10 mg/kg. Statistical Analysis of the data was carried out using a one-way analysis of variance (ANOVA) and Dennett's post hoc test for average comparison on SPSS 18.0. Mean values ± SEM (*n* = 6) were used. Significance was defined as *P* < 0.05 compared to G2. Values without an asterisk do not have significant difference compared to G2.

**Table 3:** Effect of andrographolide on liver biomarkers in hyperlipidemic rats.

	Albumin (G/L)	TB (umol/L)	AP (IU/L)	ALT (IU/L)	AST (IU/L)
Group 1	14 ± 0.37*	3 ± 0.00	60.67 ± 2.82*	58 ± 0.52*	214.6 ± 8.01*
Group 2	11.67 ± 0.21	2.5 ± 0.22	84.17 ± 0.95	65.17 ± 0.87	184 ± 3.98
Group 3	13 ± 0.00*	2.5 ± 0.22	68.83 ± 0.95*	65.83 ± 2.54	216.8 ± 3.25*
Group 4	13.33 ± 0.21*	2.5 ± 0.22	63.17 ± 1.81*	59.17 ± 0.87*	238.3 ± 12.1*
Group 5	12.67 ± 0.21*	2 ± 0.00	62.67 ± 1.93*	48.33 ± 1.8*	236.5 ± 2.43*

Albumin *F* (4,25) = 11.2, *P* < 0.001, (TB) Total bilirubin; *F* (4,25) = 4.2, *P* < 0.05, (AP) Alkaline phosphatase; *F* (4,25) = 27.4, *P* < 0.001, (ALT) Alanine aminotransferase; *F* (4,25) = 21.7, *P* < 0.001, (AST) Aspartate aminotransferase; *F* (4,25) = 9.9, *P* < 0.001.

Group (1) vehicle; Group (2) vehicle and *P. gingivalis*; Group (3) simvastatin (100 mg/kg); Group (4) andrographolide 20 mg/kg; Group (5) andrographolide 10 mg/kg. Statistical Analysis of the data was carried out using a one-way analysis of variance (ANOVA) and Dennett's post hoc test for average comparison on SPSS 18.0. Mean values ± SEM (*n* = 6) were used. Significance was defined as *P < 0.05 compared to G2. Values without an asterisk do not have significant difference compared to G2.

## References

[1] Al-Bayaty FH, Taiyeb-Ali TB, Abdulla MA, Mahmud ZB (2011). Antibacterial effects of Oradex, Gengigel and Salviathymol-n mouthwash on dental biofilm bacteria. *African Journal of Microbiology Research*.

[2] Genco RJ (1996). Current view of risk factors for periodontal diseases. *Journal of Periodontology*.

[3] Lamont RJ, Jenkinson HF (1998). Life below the gum line: pathogenic mechanisms of *Porphyromonas gingivalis*. *Microbiology and Molecular Biology Reviews*.

[4] Wang P-L, Ohura K (2002). *Porphyromonas gingivalis* lipopolysaccharide signaling in gingival fibroblasts—CD14 and toll-like receptors. *Critical Reviews in Oral Biology and Medicine*.

[5] Zhang CY, Tan B (1996). Hypotensive activity of aqueous extract of *Andrographis paniculata* in rats. *Clinical and Experimental Pharmacology and Physiology*.

[6] Zhang X-F, Tan BK-H (2000). Antihyperglycaemic and anti-oxidant properties of *Andrographis paniculata* in normal and diabetic rats. *Clinical and Experimental Pharmacology and Physiology*.

[7] Al-Bayaty F, Abdulla M, Mohamed I, Hussein S (2010). Effects of Malaysian medicinal plants on macrophage functions in vitro study. *Journal of Medicinal Plants Research*.

[8] Al-Bayaty FH, Abdulla MA, Hassan MIA, Ali HM (2012). Effect of *Andrographis paniculata* leaf extract on wound healing in rats. *Natural Product Research*.

[9] Wasman SQ, Mahmood AA, Chua LS, Alshawsh MA, Hamdan S (2011). Antioxidant and gastroprotective activities of *Andrographis paniculata* (Hempedu Bumi) in Sprague Dawley rats. *Indian Journal of Experimental Biology*.

[10] Amroyan E, Gabrielian E, Panossian A, Wikman G, Wagner H (1999). Inhibitory effect of andrographolide from *Andrographis paniculata* on PAF-induced platelet aggregation. *Phytomedicine*.

[11] Buschmann J (2013). The OECD guidelines for the testing of chemicals and pesticides. *Methods in Molecular Biology*.

[12] Cass JS, Campbell IR, Lange L (1963). A guide to production, care and use of laboratory animals. An annotated bibliography. 7. Special techniques; preparation of animals for use; handling; anesthesia, euthanasia, resuscitation; surgical techniques. *Federation Proceedings*.

[13] Gibson FC, Yumoto H, Takahashi Y, Chou H-H, Genco CA (2006). Innate immune signaling and *Porphyromonas gingivalis*-accelerated atherosclerosis. *Journal of Dental Research*.

[14] Lalla E, Lamster IB, Hofmann MA (2003). Oral infection with a periodontal pathogen accelerates early atherosclerosis in apolipoprotein E-null mice. *Arteriosclerosis, Thrombosis, and Vascular Biology*.

[15] Cutler CW, Shinedling EA, Nunn M (1999). Association between periodontitis and hyperlipidemia: cause or effect?. *Journal of Periodontology*.

[16] Draper HH, Hadley M (1990). Malondialdehyde determination as index of lipid peroxidation. *Methods in Enzymology*.

[17] Choudhary MI, Naheed S, Jalil S, Alam JM, Atta-Ur-Rahman A-U (2005). Effects of ethanolic extract of Iris germanica on lipid profile of rats fed on a high-fat diet. *Journal of Ethnopharmacology*.

[18] Woo M-N, Bok S-H, Choi M-S (2009). Hypolipidemic and body fat-lowering effects of Fatclean in rats fed a high-fat diet. *Food and Chemical Toxicology*.

[19] Bolkent S, Yanardag R, Karabulut-Bulan O, Yesilyaprak B (2005). Protective role of Melissa officinalis L. extract on liver of hyperlipidemic rats: a morphological and biochemical study. *Journal of Ethnopharmacology*.

[20] Hou Y, Shao W, Xiao R (2009). Pu-erh tea aqueous extracts lower atherosclerotic risk factors in a rat hyperlipidemia model. *Experimental Gerontology*.

[21] Mantha SV, Prasad M, Kalra J, Prasad K (1993). Antioxidant enzymes in hypercholesterolemia and effects of vitamin E in rabbits. *Atherosclerosis*.

[22] Iliskovic N, Singal PK (1997). Lipid lowering: an important factor in preventing adriamycin-induced heart failure. *American Journal of Pathology*.

[23] Del Boccio G, Lapenna D, Porreca E (1990). Aortic antioxidant defence mechanisms: time-related changes in cholesterol-fed rabbits. *Atherosclerosis*.

