# Supplementary material for: Acute Toxicity and the Effect of Andrographolide on Porphyromonas gingivalis-Induced Hyperlipidemia in Rats
Source: Biomed Res Int. 2013 Jun 13;2013:594012. doi: 10.1155/2013/594012 (PMC3703379; doi:10.1155/2013/594012)
Supplement: Supplementary file 1 — No signs of any significant abnormality were observed in renal or liver observations at doses 100 mg/kg and 500 mg/kg of andrographolide for 15 days between the control and treated groups. [file 594012.f1.docx]

## Supplementary data

## Table 1A- Effect of andrographolide on the renal function of male rats

| Dose | Sodium  (mmol/L) | Potassium  (mmol/L) | Chloride  (mmol/L) | CO_2_  (mmol/L) | Anion gap  (mmol/L) | Urea  (mmol/L) | Creatinine  (µmol/L) |
| --- | --- | --- | --- | --- | --- | --- | --- |
| Vehicle (CMC) | 139.05 ±0.56 | 4.91 ± 0.04 | 105.71 ± 0.57 | 22.75 ± 0.55 | 17.11 ± 0.58 | 5.40 ± 0.38 | 34.88 ± 2.48 |
| Andrographolide  (100 mg/kg) | 140.03 ± 1.54 | 4.89 ± 0.03 | 106.13 ± 1.03 | 23.60 ± 1.19 | 18.15 ± 0.61 | 5.42 ± 0.50 | 35.67 ± 2.35 |
| Andrographolide  (500 mg/kg) | 140.50 ± 0.76 | 4.85 ± 0.05 | 106.17 ± 0.28 | 23.56 ± 1.05 | 18.42 ± 0.45 | 6.53 ± 0.49 | 36.13 ± 3.65 |

## Values expressed as mean ± SEM. There are no significant differences between groups, significant value at P<0.05.

## Table 1B- Effect of andrographolide on the renal function of female rats

| Dose | Sodium  (mmol/L) | Potassium  (mmol/L) | Chloride  (mmol/L) | CO_2_  (mmol/L) | Anion gap  (mmol/L) | Urea  (mmol/L) | Creatinine  (µmol/L) |
| --- | --- | --- | --- | --- | --- | --- | --- |
| Vehicle (CMC) | 141.35±0.410 | 4.46 ±0.12 | 106.48±0.67 | 24.41±0.47 | 18.21±0.36 | 8.18±0.35 | 41.89±2.75 |
| Andrographolide (100 mg/kg) | 142.31 ±0.68 | 4.85 ±0.16 | 107.53±0.68 | 23.55±0.45 | 17.42±0.41 | 7.91±0.24 | 41.24±4.76 |
| Andrographolide (500 mg/kg) | 142.74± 0.47 | 4.94 ±0.08 | 107.13±0.63 | 23.51±0.78 | 18.38±0.49 | 7.93 ±0.69 | 42.15 ±2.15 |

## 1

## Values expressed as mean ± SEM. There are no significant differences between groups, significant value at P<0.05.

## Table 1C- Effect of andrographolide on liver function of male rats

| Dose | Total protein (g/L) | Albumin (g/L) | Globulin (g/L) | TB (µmol/L) | CB (µmol/L) | AP  (IU/L) | ALT  (IU/L) | AST  (IU/L) | GGT  (IU/L) |
| --- | --- | --- | --- | --- | --- | --- | --- | --- | --- |
| Vehicle (CMC) | 61.43 ±1.13 | 9.03 ±0.53 | 51.39 ±1.33 | 2.79 ±0.17 | 1.12 ±0.31 | 156.14 ±7.31 | 48.81 ±1.71 | 160.53  ± 7.17 | 4.27 ±0.16 |
| Andrographolide (100 mg/kg) | 60.37 ±1.18 | 8.56 ±0.33 | 50.23 ±0.69 | 2.82 ±0.15 | 1.31 ±0.21 | 155.21 ±6.24 | 47.02 ±1.33 | 163.18  ± 6.84 | 4.57 ±0.11 |
| Andrographolide  (500 mg/kg) | 61.23 ±1.29 | 8.12 ±0.47 | 51.11 ±1.05 | 3.08 ±0.10 | 1.02 ±0.04 | 155.13 ±8.09 | 49.52 ±1.16 | 161.87  ± 8.15 | 4.21 ±0.14 |

## TB: Total bilirubin; CB: Conjugated bilirubin; AP: Alkaline phosphatase; ALT: Alanine aminotransferase; AST: Aspartate aminotransferase; GGT: Gamma-glutamyltransferase. Values expressed as mean ± SEM. There are no significant differences between groups, significant value at P<0.05.

## Table 1D- Effect of andrographolide on the liver function of female rats

| Dose | Total protein (g/L) | Albumin (g/L) | Globulin (g/L) | TB (µmol/L) | CB  (µmol/L) | AP  (IU/L) | ALT  (IU/) | AST  (IU/L) | GGT  (IU/L) |
| --- | --- | --- | --- | --- | --- | --- | --- | --- | --- |
| Vehicle  (CMC) | 63.21 ±1.37 | 10.17 ±0.51 | 54.46 ±1.23 | 1.68 ±0.00 | 1.31 ±0.51 | 121.61 ±5.13 | 44.15 ±1.86 | 181.29  ± 5.56 | 3.42 ±0.43 |
| Andrographolide (100 mg/kg) | 64.37 ±1.46 | 11.22 ±0.61 | 55.02 ±1.42 | 1.85 ±0.00 | 1.24 ±0.48 | 124.13 ±4.48 | 45.17 ±1.63 | 182.08 ±5.65 | 3.47 ±0.41 |
| Andrographolide (500 mg/kg) | 64.62 ±1.54 | 11.14 ±0.57 | 56.34 ±1.51 | 1.73 ±0.00 | 1.20 ±0.42 | 123.16 ±5.66 | 44.13 ±1.79 | 180.81 ±5.83 | 3.85 ±0.44 |

## TB: Total bilirubin; CB: Conjugated bilirubin; AP: Alkaline phosphatase; ALT: Alanine aminotransferase; AST: Aspartate aminotransferase; GGT: Gamma-glutamyltransferase. Values expressed as mean ± SEM. There are no significant differences between groups, significant value at P<0.05.
